# Home management of lower limb lymphoedema with an intermittent pneumatic compression device: a feasibility study

**DOI:** 10.1186/s40814-019-0496-4

**Published:** 2019-09-30

**Authors:** Nyree Dunn, E. Mark Williams, Michelle Fishbourne, Gina Dolan, Jane H. Davies

**Affiliations:** 1 Faculty of Life Sciences, University of South Wales, Lower Glyntaff Campus, Room AB034, Treforest, Pontypridd, CF37 4BD UK; 2 Dewi Sant Hospital, Cwm Taf University Health Board, Pontypridd, UK; 3 Centre for Trials Research, Cardiff University, Cardiff, UK

**Keywords:** Lymphoedema, Intermittent pneumatic compression, Leg volume

## Abstract

**Background:**

Lymphoedema is a chronic condition that causes swelling in the body tissues. Presently, there is no cure for lymphoedema; instead, current treatment is aimed at lifelong management to help control symptoms. Intermittent pneumatic compression (IPC) therapy can be considered as an adjunct to standard lymphoedema care; however, research regarding the efficacy of this treatment modality is limited.

**Methods:**

Twenty participants were recruited from an outpatient lymphoedema clinic (South Wales, UK) to a feasibility randomised control trial designed to evaluate the efficacy of an IPC device (LymphAssist, Huntleigh Healthcare) in reducing lower limb volume. The primary objective was to assess feasibility in terms of (1) study feasibility, including recruitment, retention and assessment of outcome measures, and (2) intervention feasibility, including intervention fidelity and acceptability to participants. Participants were randomly assigned to a control group (*n* = 10) or intervention group (*n* = 10). The control group received their standard lymphoedema care only for a 6-month period, whereas the intervention group received their standard lymphoedema care plus an IPC device to use for 6 months. A bilateral lower limb assessment and quality of life survey were undertaken at baseline and 3- and 6-month time points.

**Results:**

The study recruited to target within the planned time frame with a retention rate of 80%. Issues relating to potential recruitment bias and study attrition were identified and possible solutions explored. In addition, supplementary primary outcome measures that are important to the study population were identified and will be incorporated into the design of future studies.

**Conclusion:**

This feasibility study identified that a larger randomised controlled trial investigating the efficacy of home use IPC devices is feasible with modifications to the study protocol.

**Trial registration:**

This trial is registered with clinicaltrials.gov (NCT03825263).

## Introduction

Lymphoedema is a chronic condition that causes swelling in the body tissues due to an excess accumulation of protein-rich fluid called lymph. This occurs as a result of lymphatic failure which can be genetic in origin (primary lymphoedema) or a consequence of damage to the lymphatics usually by trauma, inflammation and damage of the lymph nodes (secondary lymphoedema) [1]. It can affect any part of the body but usually occurs in the arms or legs and has an estimated prevalence of between 2.29 and 3.59 cases per 1000 of the general population in the UK [2].

Key characteristics of lymphoedema include an increase in limb size and skin changes, as well as increased limb heaviness and pain, which have negative sequelae for both physical and psychosocial health [3]. Presently, there is no cure for lymphoedema; instead, current treatment is aimed at lifelong management to help control symptoms. Such treatment is based on decongestive lymphatic therapy (DLT) which is a combination of manual lymph drainage (MLD), compression therapy, exercises and skin care; this is accepted internationally as the gold standard for successful lymphoedema management [4, 5].

Intermittent pneumatic compression (IPC) therapy can be considered as an adjunct for DLT. IPC devices consist of pneumatic cuffs that are incorporated into a compression garment, which is connected to a pump and applied to the limb. Multiple-chamber garments typically provide sequential compression in an ascending pattern up the limb. Pumps vary in timing cycles and amount of pressure produced, ranging from low-pressure, slow-inflation to high-pressure, rapid-inflation devices [6].

Research regarding the efficacy of IPC is limited. Whilst early studies suggested that MLD and IPC are equally as effective in reducing upper limb oedema [7], studies examining sequential IPC as a treatment for lower limb lymphoedema [1, 6, 8, 9] demonstrated a lack of consensus with regard to treatment parameters. Historically, IPC pumps have used sequential cycles to provide a peristaltic massaging effect along the limb towards its root. This, however, does not mimic the MLD process, which starts with the unaffected lymph nodes and region of the body and moves proximally to distally [10]. MLD is a specialised massage technique that helps stimulate the lymphatic system and encourages the flow of lymph fluid. However, it can be both cost and time intensive for clinicians and patients alike, and it is not always accessible due to an insufficient number of trained therapists [11]. Newer IPC devices have been designed to mimic MLD but have not yet been evaluated in research studies. Furthermore, no studies have examined IPC as a home treatment. Hence, several aspects of IPC as a treatment for lymphoedema require evaluation in the form of robust clinical research.

This feasibility study aimed to evaluate a proposed methodology designed to assess the efficacy of IPC for the treatment of lower limb lymphoedema. The study methodology consisted of a pilot randomised control trial of IPC plus standard lymphoedema care versus standard lymphoedema care alone. Specific objectives were to determine (1) study feasibility, including recruitment, retention and assessment of outcome measures, and (2) intervention feasibility, including intervention fidelity and acceptability to participants.

## Methods

### Study design

This feasibility study was a non-blinded, randomised, controlled trial (RCT). Ethical approval for this study was obtained from the appropriate local NHS Research Ethics Committee (LREC No 17/WA/0076) and registered with ClinicalTrials.gov, NCT03825263 (https://clinicaltrials.gov/ct2/show/NCT03825263?term=IMPRESS&rank=5). Study duration was 6 months for each participant, with follow-up appointments at 3-month and 6-month time points. This time frame was chosen as it fitted with the routine appointments at the lymphoedema clinic where the study was undertaken.

### Participants

**Fig. 1:**
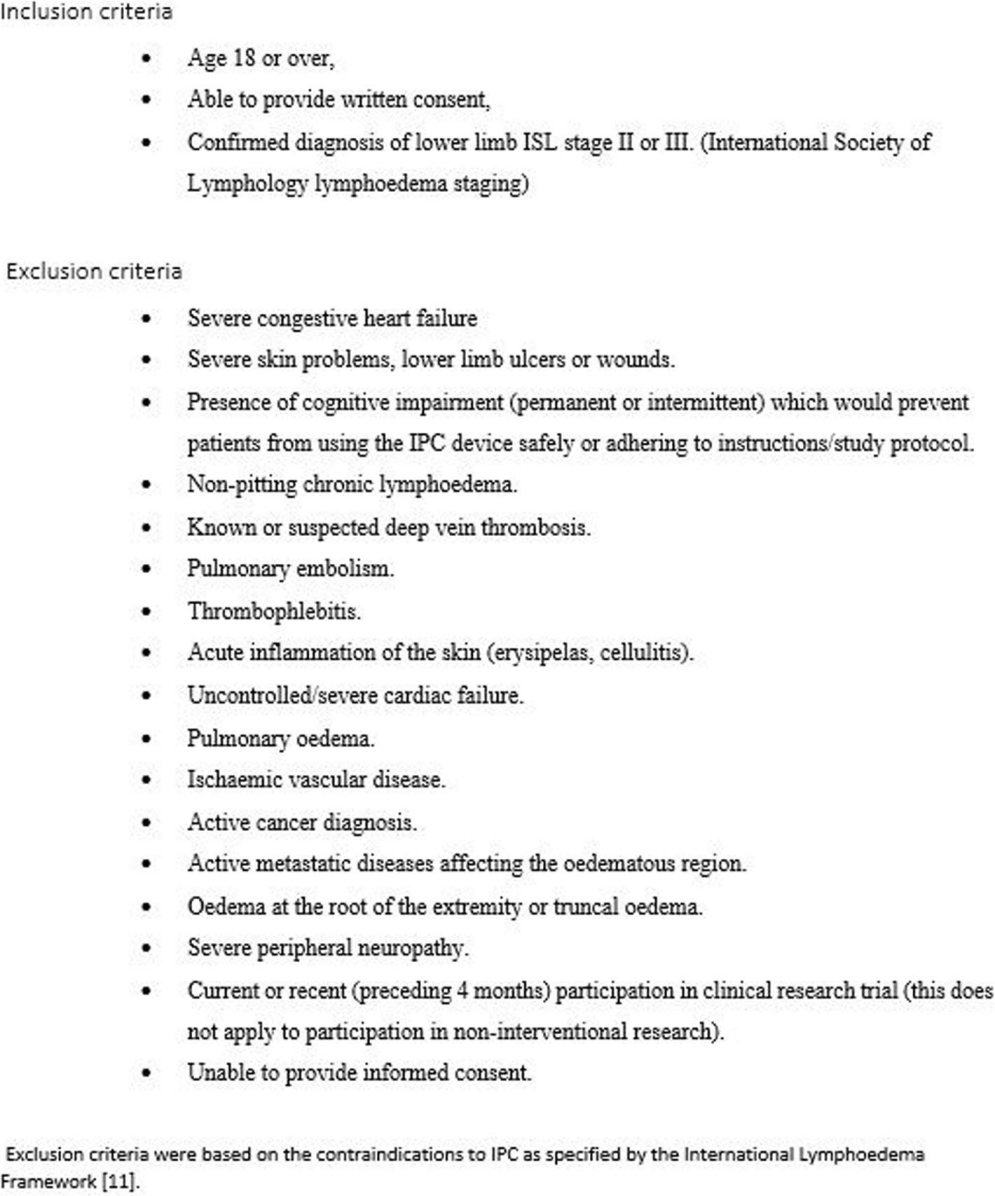
Inclusion and exclusion criteria

Preliminary screening was carried out by the clinical lead of the outpatient lymphoedema clinic who notified the study investigators of any eligible and willing patients. A sample of twenty participants who met the study inclusion and exclusion criteria (Fig. 1) and agreed to participate were recruited. All participants provided written consent.

### Outcome measures

As lymphoedema is defined by an increase in limb size, the primary outcome measure used in this study was change in limb volume. This parameter is universally used in the assessment of lymphoedema treatments; results would therefore allow comparison with existing research/literature. Secondary outcomes included change in quality of life scores according to the Quality of Life Enjoyment and Satisfaction Questionnaire short form (Q-LES-Q-SF) [12] and usability of the IPC device.

### Baseline assessment

Following consent, each participant underwent a baseline assessment to record the following parameters: age, gender, height and weight (Table 1). Further information about the type of lymphoedema and its stage according to the International Society of Lymphology (ISL) staging [5] as well as a brief medical history was obtained by reviewing the participants’ medical notes. Next, bilateral limb assessment was undertaken which included leg volume measurement using a circumferential tape measure method; a non-stretch tape measure (Medi, Germany) was used to measure leg circumference at 40-mm intervals from the top of the malleolus to a significant clinical end point that was taken from the patient’s medical notes. To assure consistent measurement, the same registered nurse with experience in circumferential leg measurements using a tape measure performed the measurements, at baseline and at any follow-up appointments. The leg circumference measures were used to calculate the volume of the 40-mm leg segments using simple software (LymCalc V 4.0, UK) where leg volumes are calculated by adding together segments [13]. Participants also completed the Quality of Life Enjoyment and Satisfaction Questionnaire short form (Q-LES-Q-SF) [12] at baseline, prior to being randomly assigned to the control group (*n* = 10) or the intervention group (*n* = 10).

**Table 1:** Baseline demographics

	Control (*n* = 10)	Intervention (*n* = 10)	All (*n* = 20)
Mean age (years) (SD)	41.3 ± 13.2	58.3 ± 11.5	49.8 ± 14.9
Gender (M to F) (%)	50:50	10:90	30:70
Weight (kg) (SD)	92.9 ± 29.2	95.5 ± 43.8	94.2 ± 36.2
Height (cm) (SD)	169.6 ± 8.5	162.9 ± 10.4	166 ± 9.8
BMI (SD)	30.2 ± 8.4	36.2 ± 12.2	33.2 ± 10.6
Lymphoedema stage			
II	100% (10)	100% (10)	100% (20)
III	0% (0)	0% (0)	0% (0)

### Randomisation

Participants were randomly assigned to the control or intervention group via the use of sequentially numbered, opaque, sealed envelopes. The envelopes were sealed by an independent administrator and concealed from the study investigator until recruitment, numbers one to ten indicated the control group and 11 to 20 indicated the intervention group. This simple method of randomisation creates a low risk of bias [14].

### Control group

Participants in the control group received their standard care for the 6-month study period. Standard prescribed care within the lymphoedema clinic consisted of the four components of DLT; however, it was recognised at the study outset that this could potentially differ amongst participants for a number of reasons. Firstly, the care is largely self-administered with patients choosing whether or not to implement the recommended components of the treatment regimes. Secondly, the prescribed care can vary; for example, some patients, although not all, are prescribed MLD sessions with a trained therapist. In order to capture this information, participants in the control group were asked to complete weekly treatment diaries which were returned when they attended their 3- and 6-month reviews.

### Intervention group

**Fig. 2:**
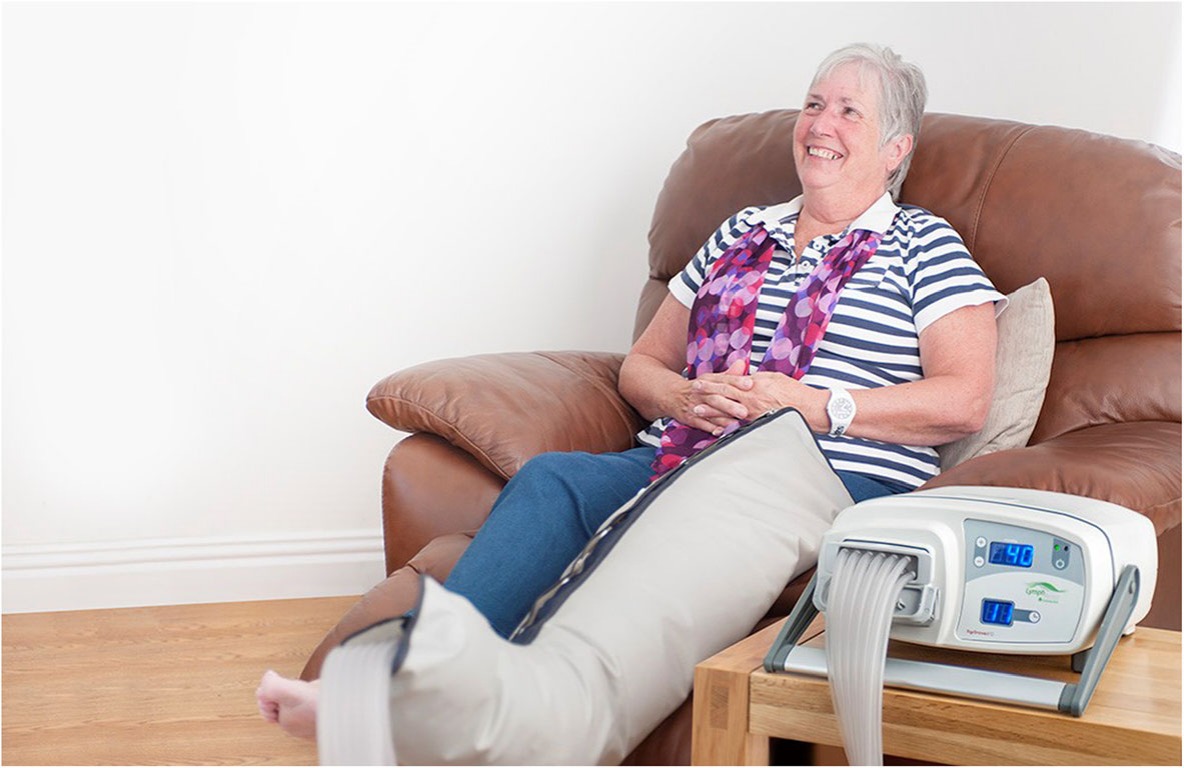
IPC device in use (LymphAssist, Huntleigh Healthcare)

The intervention group received their standard care plus an IPC device (LymphAssist, Huntleigh Healthcare) (Fig. 2) to use for 6 months in addition to receiving their standard lymphoedema care. The IPC devices operated on a programme designed to mimic the MLD process, first applying compression to the proximal aspect of the limb before progressing distally. For safety purposes, the devices were set to operate at a pressure of 40 mmHg which is recommended by the device manufacturer for home use of the device; this is low in comparison to the pressures used in typical IPC devices which can reach up to 120 mmHg. Each participant was given a demonstration by the study investigator on how to operate the device at their clinic appointment, before trialling the equipment themselves. Each device included an operating instruction manual, and participants were advised to contact the study team if they had any issues or queries related to using the device. Participants were instructed to use the IPC device twice a day (preferably morning and evening) for 35-min cycles (as recommended by the device manufacturer). Diaries were also provided for the intervention group to record their weekly treatment regimens and IPC device use. Participants in the intervention group were also asked to complete a brief questionnaire that was designed to assess the usability of the IPC device. The questionnaire involved the participants rating the comfort, ease of use and practicality of the device using a visual analogue scale and was completed at 3- and 6-month time points.

### Follow-up assessments

All baseline assessments including limb volume measurements and quality of life surveys were repeated at 3- and 6-month time points.

## Results

### Recruitment

The study recruited to target within the planned 6-week time frame. A screening log was not completed so it was not possible to gain information regarding patients who were (i) ineligible or (ii) eligible to participate but declined. Despite patients with stage III lymphoedema being eligible to participate in the study, the study population was made up exclusively of participants with stage II lymphoedema.

### Retention

**Fig. 3:**
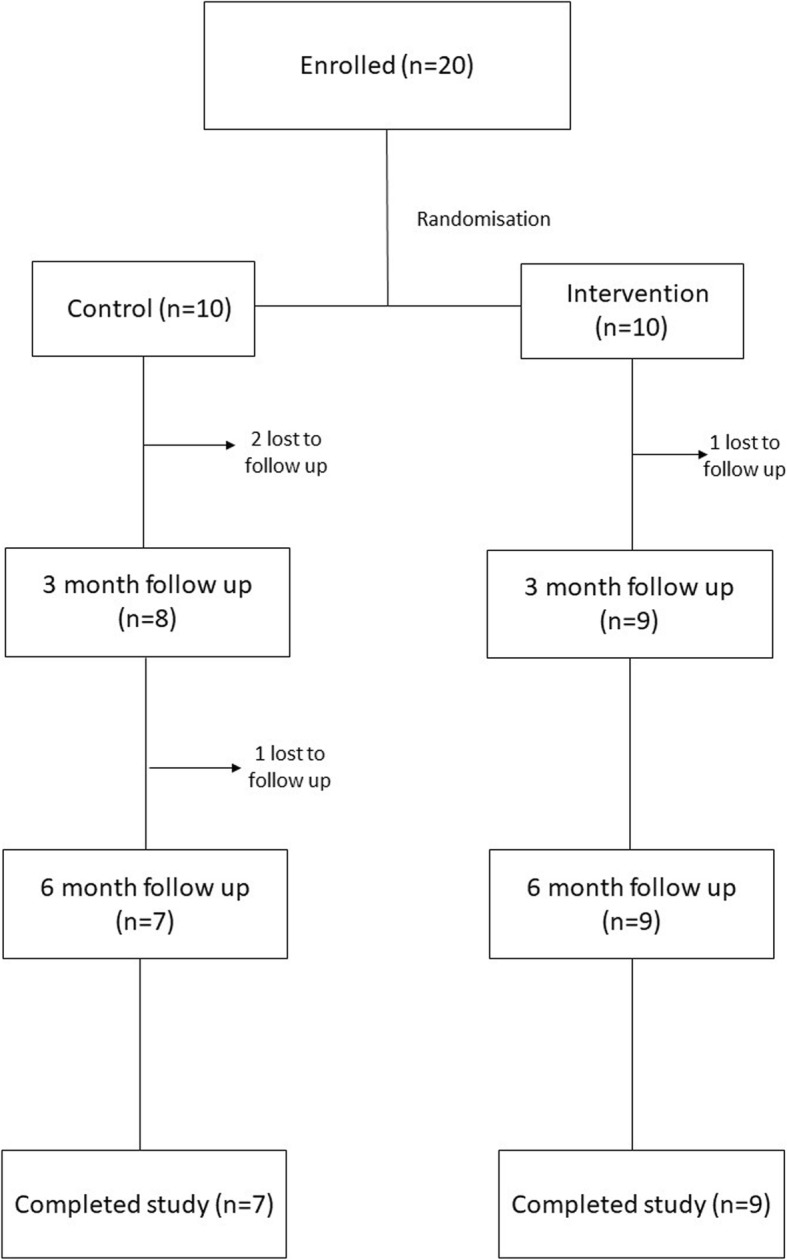
Participant flow

The study achieved a retention rate of 80% of participants. Four participants withdrew from the study, three at 3 months and one at 6 months (Table 2) (Fig. 3). Three withdrew from the control group and one withdrew from the intervention group after finding the IPC device too uncomfortable to continue using.

**Table 2:** Demographics of participants who completed the study vs. those who were lost to follow-up

	Completed study (*n* = 16)	Lost to follow-up (*n* = 4)
Mean age (years) (SD)	52.1 ± 10.9	40.2 ± 25.3
Gender (M to F) (%)	20:60	10:10
Weight (kg) (SD)	97.2 ± 38.2	82.3 ± 28.4
Height (cm) (SD)	168.1 ± 10.6	164.2 ± 8.1
BMI (SD)	33.9 ± 10.9	30 ± 7.7
Lymphoedema stage		
II	100% (16)	100% (4)
III	0% (0)	0% (0)
Randomisation group		
Control	70% (7)	30% (3)
Intervention	90% (9)	10% (1)

### Adherence to study protocol

Although participants were given a demonstration on how to complete their weekly treatment diary at their baseline visit and also had an instruction page to refer to, completion of the diaries was found to be poor in both the control and intervention group. Participants were asked to estimate how many minutes they spent each day on each of the four aspects of DLT and using the IPC device (if applicable); however, this was infrequently done, with several participants giving a total weekly estimate instead. Furthermore, missing diary data was also problematic (control group, 37% at 3 months and 42% at 6 months; intervention group, 20% at 3 months and 33% at 6 months).

Of the diaries returned by the intervention group, 75% (6/8) reported using the IPC device twice daily as recommended by the manufacturers at the 3-month review point; this figure had decreased to 66% (4/6) by the 6-month review.

## Acceptability of intervention

The usability questionnaire showed good acceptance of the study intervention; from the nine participants that completed the IPC usability questionnaire, an average score of 57/60 was returned at the 3-month time point and 58/60 at the 6-month time point.

### Outcomes measures

Anthropometric measures between the control and intervention groups were similar; although there was a greater proportion of females in the intervention group than in the control group (90% vs 50% respectively) (Table 1). The overall gender ratio of the study population was male to female = 30:70; this reflects the normal distribution of the condition which is more commonly seen in females [15].

Both the control group and the intervention group showed a decrease in mean limb volume at both 3- and 6-month time points (Table 3). Mean QOL scores (according to Q-LES-Q-SF) in both the control and intervention decreased slightly as the study progressed (Table 4).

**Table 3:** Mean changes in affected limb volume

Time Period	Control (ml)	Intervention (ml)	Difference in mean limb volume change (control–intervention) (ml)
0–3 months	− 360 95% CI [− 593, − 128]	− 78, 95% CI [− 437, 279]	− 282 (*p* = 0.13)*
0–6 months	− 513 95% CI [− 1065, 39]	− 530 95% CI [− 1100, 39]	17 (*p* = 0.56)*
3–6 months	− 153 95% CI [− 667, 362]	− 451 95% CI [− 1125, 223]	298 (*p* = 0.74)*

*P values from Mann-Whitney *U* test

**Table 4:** Mean quality of life scores ± SD

	Control	Intervention	All
Baseline	50 ± 17	48 ± 7	49 ± 12
3 months	51 ± 17	46 ± 11	48 ± 14
6 months	49 ± 16	46 ± 7	47 ± 11

## Discussion

### Study feasibility

The study recruited to target in a relatively short time frame hence suggesting that recruitment to a larger study of similar design would be attainable. However, it was noted by the study investigator that most participants had stage II lymphoedema with well-controlled symptoms and were highly concordant with the treatment regimens prescribed by the clinic. According to the ISL, lymphoedema stage II consists of limb swelling which is not reduced by elevation, pitting may or may not be present (if fibrosis is present); stage III lymphoedema consists of hard tissue, increased skin folds, fat deposits and warty overgrowths [5]. It is possible that patients with stage III lymphoedema were excluded from the study on account of them being more likely to have severe skin problems or lower limb ulcers/wounds which are contraindications to the use of IPC as specified by the ISL [5]. Alternatively, recruitment bias is a possibility, with the study being more likely to recruit patients who were highly motivated to self-manage their condition and as a result had a well-controlled lymphoedema status. However, unfortunately, it was not possible to assess this further as a screening log was not completed by clinic staff. This can perhaps be partly attributed to the fact that the study was undertaken in a research naïve environment. Future studies undertaken in this setting would therefore require a higher level of support for clinic staff. It is vitally important to recognise and address recruitment bias because it can significantly affect the integrity of randomised controlled trials [16] with a reduction in external validity hence meaning the results would not be representative of the general lymphoedema population. The current research aims to assess the efficacy of IPC across both stage II and stage III lymphoedema; it is therefore paramount that this issue is addressed in future planned studies. Strategies to ensure effective recruitment could include designing a clear recruitment strategy prior to study initiation, having more flexible participation hours along with a less complex protocol. Allowing sufficient time and staffing can also help minimise recruitment bias [17].

Attrition is expected in most if not all clinical trials; however, bias can be expected with the attrition rate exceeds 20% [18]. The overall study attrition rate of 20% (control group, 30%; intervention group, 10%) was therefore concerning. Attrition can undermine the internal and external validity of a study, often resulting in biased findings, especially if the participants are not lost at random and have certain characteristics that could affect the outcome. Analysis of the dataset did not reveal any factors significantly associated with attrition in this study. However, it is suspected that lack of interest, particularly from the control group, was a key contributing factor to attrition in this feasibility study. Many of the control group expressed their disappointment at being not being allocated to receive the IPC device, and this could have influenced their commitment to participation and attendance at follow-up appointments. Additional contributing factors, which are commonly cited in the literature, could have included time restraints and changes to daily life [19]. In order to try and address this apparent apathy within the control group, it has been decided that our next study will incorporate a cross over design, where participants will act as their own controls for the first 5 weeks of their participation before being allocated an IPC device to use for the subsequent 5 weeks.

Issues associated with participant burden are often closely associated with attrition. Study duration, intenseness and invasiveness of the procedure all have an effect on the participant and need to be considered when planning a larger scale study [20]. The 6-month duration of this study was, in retrospect, too long and probably represented a considerable source of participant burden. Although participants needed to attend only three clinic appointments during this time, they were still required to complete daily diaries and use the IPC device twice daily (for those in the intervention group). In order to try and address this issue, the timescale of our next study will be based on the treatment lengths seen in clinical practice. Typically, a lymphoedema patient receiving manual lymphatic drainage from a therapist will undergo therapy for around 3–4 weeks [11]; hence, it has been decided that participants should receive the IPC intervention for a similar time period. Reducing the length of the treatment periods is intended to increase the likelihood of the participant adhering to the treatment regime for the treatment duration, which could have important implications for the results of future studies.

Data collection via the treatment diaries was adversely affected by poor completion rates and lack of adherence to the diary completion instructions. Although a cut-off point has not been established in regards to missing data, more than 10% can lead to a bias analysis [21]. In addition to these problems, which according to the literature are common-place, additional issues relating to ‘back-filling’ of diary entries can also adversely affect the quality and reliability of the data collected [22]. A study by Stone et al. [22] found much better diary completion compliance when using electronic diaries as opposed to paper diaries (94% vs. 11% respectively); however, other studies have not replicated this finding [23]. Whilst financial limitations would rule out the use of electronic diaries for our subsequent study, the paper data collection diaries will be reviewed with a view to simplifying them and making their completion less onerous.

The primary outcome measure assessed within this study was limb volume change. This study showed that the IPC device had no effect on limb volume over the 6-month period (as indicated by 95% confidence intervals); however, it could be that the study was underpowered to detect such an effect. Variation in standard lymphoedema care across both the intervention group and control group was noted; those participants receiving MLD during the study duration typically displaying at least temporary reductions in affected limb volume. Similarly, one participant in the control group lost more than 7 kg in weight during their participation in the study as a result of being on a calorie-controlled diet hence meaning that any reduction in limb volume could be attributed to this. Such confounding factors will be taken into consideration when undertaking a sample size calculation for future studies.

Whilst volume change as a parameter was successfully measured in this study, informal discussions with participants revealed that other factors, such as perceived ‘limb heaviness’ and ‘tightness’, were equally or more important to them when assessing improvements or deterioration of their condition. Hence, it was decided that our subsequent study should include both objective and subjective measures aimed at assessing such factors. These parameters will be measured objectively using the Myoton Pro, which works by recording damped natural oscillation of soft biological tissue in the form of an acceleration signal, providing outputs such as state of tension, biomechanical and viscoelastic properties [24]. Participant perceived limb tightness and heaviness will be also assessed subjectively via the use of visual analogue scales.

The use of the Q-LES-Q-SF showed little or no changes in scores at 3 months or 6 months; it cannot be determined if this was a true representation of the participants’ quality of life or if the questionnaire used was not sensitive enough to assess aspects of quality of life that specifically relate to lymphoedema. In view of this, a questionnaire that has been designed and validated for this purpose, the Lymphoedema functioning, disability and Health Questionnaire for Lower Limb Lymphoedema (Lymph-ICF-LL), will be utilised for our subsequent study. The questionnaire has five domains: physical function, mental function, general tasks/household activities, mobility activities and life domains/social life. These domains were identified after a thorough literature review focusing on problems in functioning related to the development of lower limb lymphoedema [25].

### Intervention feasibility

Assessment of intervention fidelity was hindered by the poor rate of diary return. Available data suggest that use of the IPC device as recommended by the manufacturers was largely adhered to, although this decreased from the three to 6-month time points, again suggesting issues associated with participant burden. The acceptability questionnaire demonstrated excellent acceptance of the intervention across all three parameters of comfort, ease of use and practicality.

## Conclusion

This feasibility study has identified several important issues that require consideration in the design of a larger-scale randomised control trial which will investigate the efficacy of IPC in the treatment of lymphoedema. Specifically, potential issues relating to recruitment bias and study attrition have been identified and possible solutions explored. In addition, supplementary primary outcome measures that are important to the study population have been identified and will be incorporated into the design of future studies. Whilst this feasibility study utilised a new generation IPC device which operated on a programme designed to mimic the MLD process (proximal to distal compression), there is also a need to evaluate the efficacy of the more commonly used, distal to proximal compression modes. Our planned future study will therefore randomise participants to utilise the IPC device on one of these compression modes for the intervention period of the study.

## Data Availability

The data sets analysed during this current study are available from the corresponding author on reasonable request (nyree.dunn@southwales.ac.uk).

## Abbreviations

DLT: Decongestive lymphatic therapy
IPC: Intermittent pneumatic compression
ISL: International Society of Lymphology
Lymph-ICF-LL: Lymphoedema functioning, disability and Health Questionnaire for Lower Limb Lymphoedema
MLD: Manual lymphatic drainage
Q-LES-SF: Quality of Life Enjoyment and Satisfaction Questionnaire short form
RCT: Randomised controlled trial
